# Increased circulating Th17 cells and altered CD4 T cell maturation and differentiation in active tuberculosis with type 2 diabetes: a pilot study

**DOI:** 10.3389/fimmu.2025.1637868

**Published:** 2025-09-09

**Authors:** Paul Ogongo, Yoscelina E. Martinez-Lopez, Anthony Tran, Cecilia S. Lindestam Arlehamn, Alessandro Sette, Ilse A. Dominguez-Trejo, Lizette Garza, America M. Cruz-Gonzalez, Raul Loera-Salazar, Javier E. Rodríguez-Herrera, Genesis P. Aguillón-Durán, Esperanza M. Garcia-Oropesa, Joel D. Ernst, Blanca I. Restrepo

**Affiliations:** ^1^ Division of Experimental Medicine, University of California, San Francisco, San Francisco, CA, United States; ^2^ School of Public Health, University of Texas Health Science Center at Houston, Brownsville, TX, United States; ^3^ Center for Vaccine Innovation, La Jolla Institute for Immunology, La Jolla, CA, United States; ^4^ Department of Infectious Disease and Immunology, Center for Vaccine Research, Statens Serum Institut, Copenhagen, Denmark; ^5^ Department of Medicine, Division of Infectious Diseases and Global Public Health, University of California, San Diego, La Jolla, CA, United States; ^6^ Department of Health and Biomedical Sciences, University of Texas Rio Grande Valley, Edinburg, TX, United States; ^7^ Departamento Estatal de Micobacteriosis, Secretaría de Salud de Tamaulipas, Reynosa/Matamoros/Ciudad Victoria, Tamaulipas, Mexico; ^8^ Unidad Académica Reynosa-Aztlán, Universidad Autónoma de Tamaulipas, Reynosa, Mexico; ^9^ Department of Human Genetics, South Texas Diabetes and Obesity Institute, School of Medicine, University of Texas Rio Grande Valley, Edinburg, TX, United States; ^10^ Population Health and Host Pathogens Interactions Programs and International Center for the Advancement of Research & Education (I.CARE), Texas Biomedical Research Institute, San Antonio, TX, United States

**Keywords:** Th17 cell subsets, tuberculosis, type 2 diabetes, dysregulated, T cell maturation

## Abstract

**Introduction:**

Type 2 diabetes (T2D) is a major risk factor for developing tuberculosis (TB). However, understanding the role of defective T cell responses in T2D and TB has been difficult, largely due to inconsistencies across studies. These discrepancies often stem from T cell subset classification primarily relying on cytokine expression profiles, which may not fully capture the complexity of T cell maturation, differentiation, and function in TB patients with T2D.

**Objective and methods:**

In this pilot study, we sought to identify alterations in phenotypic and ex vivo responses of CD4 T cells to *Mycobacterium tuberculosis* (Mtb) antigens in people with TB with or without T2D. We evaluated peripheral blood mononuclear cells (PBMC) by high-parameter spectral flow cytometry and assessed T cell differentiation using a cytokine agnostic approach based on validated cell surface markers expression.

**Results:**

We found major alterations in specific CD4 T cell properties by T2D status, despite no difference in the frequency of bulk CD4 or CD8 T cells. TB-T2D patients (*vs* TB alone) had fewer circulating naïve CD4 T cells, higher frequency CD4 T cell responses to Mtb antigens, and increased circulating Th1 and three subsets of Th17 cells. Multivariable analysis confirmed that T2D was independently associated with these alterations in maturation state, differentiation phenotype, and the activation of Mtb antigen-responsive CD4 T cells.

**Conclusion:**

This pilot study reveals CD4 T cell alterations in T2D that likely worsen TB outcomes. A reduced naïve CD4 T cell pool, increased central memory and antigen-activated CD4 T cells, and elevated Th1 and three Th17 cell subsets suggest a pro-inflammatory environment favoring responses that may promote, rather than control TB. These findings highlight immune dysfunctions that could be targeted by host-directed therapies to prevent TB and improve outcomes in T2D patients.

## Introduction

Tuberculosis (TB) and Type 2 diabetes (T2D) are both global health priorities, with TB affecting 10 million people annually and T2D estimated to have affected 10.5% of the adult population with a projected rise to 12.2% by 2045 (1). Consequently, TB and T2D affect some of the same individuals with T2D worsening TB outcomes. T2D has been shown to alter immune function, increasing the risk for other diseases (2, 3). Chronic hyperglycemia, a hallmark of T2D, compromises innate immune cell phenotypes and functions (e.g. phagocytosis, antigen processing and presentation to T cells, and bactericidal activities) (4–6). T2D has also been associated with altered cell-mediated immune function, including T cell differentiation, reduced expression of migration-associated chemokine receptors on T cells, and impaired migration of effector memory T cells (7).

T2D increases TB risk more than 3-fold (8) and is also associated with TB treatment failure (9, 10), but the mechanisms underlying the increased risk and severity are incompletely understood. CD4 T cells play a critical role in mediating responses essential for controlling initial *Mycobacterium tuberculosis* (Mtb) infection and limiting bacterial growth (11–15). In TB patients, high blood glucose is associated with alterations in T cell activation, differentiation, and cytokine secretion, but the results of individual studies are contradictory. Some studies found reduced secretion of Th1 cytokines such as IFNγ and TNFα (16–18), others reported higher Th1 cytokine responses (19, 20), while still others reported no difference in IFNγ, IL2, and TNFα in TB-T2D patients versus TB alone (21, 22). Hence, it is unclear whether Th1-associated responses are higher, similar, or lower in T2D-TB compared to TB patients without T2D. Differences between study populations, experimental designs, and failure to control for potential confounders during data analysis may explain these contrasting findings.

Beyond Th1 cells, emerging evidence indicates that Th17 cells can contribute to the control of Mtb in humans (23–25). In Mtb-infected individuals, diminished Th17 responses are associated with progression to TB disease (23, 24). In addition, Th17-like responses to Mtb antigens were enriched in Mtb-exposed individuals who remained TST and IGRA-negative compared to IGRA-positive controls (25). Mtb-responsive Th17 cells are enriched in Mtb-infected human lungs compared to matched blood and inversely associated with plasma IL-1β (26). However, in people with TB, Th17 cells have also been associated with the severity of disease (27). In T2D, the levels of peripheral IL17-producing cells are reduced in latent TB (28) but are significantly elevated in patients with TB-T2D compared to TB (20, 22). However, human Th17 cells are heterogeneous, including subsets that differ in the ability to produce IL17 to Mtb antigen stimulation (29). Furthermore, Th17 cells respond preferentially to distinct Mtb antigens (30). In contrast to a protective role of Th17 cells in certain infections, Th17 cell responses can be pathogenic in certain inflammatory diseases, including psoriasis, rheumatoid arthritis, inflammatory bowel diseases, and multiple sclerosis (31, 32). Hence, there is a need to understand the role of Th17 cells in TB control or pathology in TB-T2D patients.

In this pilot study, we sought to address gaps in understanding the alterations in phenotype and *ex vivo* responses to Mtb antigens by CD4 T cells from TB patients with T2D. We performed a high-parameter analysis of CD4 T cells by spectral flow cytometry on a well-characterized cohort of newly diagnosed active TB patients with or without T2D, and conducted multivariable analysis to assess the independent contribution of T2D to the observed alterations. We found that whereas a chronic history of T2D did not affect the overall frequency of circulating bulk CD4 or CD8 T cells, there were distinct alterations in specific CD4 T cell properties, including maturation state, differentiation, and the activation of Mtb antigen-responsive CD4 T cells.

## Methods

### Participant enrollment and characterization

Adults with newly diagnosed TB were enrolled at referral TB clinics in Reynosa and Matamoros, Mexico. Exclusion included age older than 60 years, HIV-positive, reported alcohol/drug abuse, or type 1 diabetes. Enrollment procedures followed guidelines from the Institutional Review Boards in Mexico (110/2018/CEI) and the United States (HSC-SPH-19-0308; HSC-SPH-14-1007), and participants signed informed consent. TB diagnosis was based on isolation of Mtb or a positive smear for acid-fast bacilli and an abnormal chest x-ray. Body-mass index and waist:hip ratio (WHR) were documented as before (33). T2D was based on hyperglycemia (fasting glucose ≥126 mg/dL or random ≥200 mg/dL or HbA1c ≥ 6.5%). Estimates of insulin resistance and % beta-cell function used the homeostatic model assessments HOMA1-IR and HOMA2, respectively (33, 34), on participants with fasting blood glucose and no insulin use.

### PBMC antigen stimulation

PBMC isolated from heparinized peripheral blood were cryopreserved in liquid nitrogen. Cells were thawed and transferred into R10 media (RPMI 1640 containing L-glutamine with 10% FBS, 1% PenStrep, and 1% Hepes). Cells were centrifuged at 900g for 5 minutes, resuspended in 5 ml R10, transferred to a 6-well plate, and rested overnight at 37°C/5% CO_2_. Cells were counted and 1×10^6^ live cells in 200 μL of R10 were stimulated with Mtb peptide mega pool (Mtb300) (2μg/ml) or staphylococcal enterotoxin B (SEB) positive control (1μg/ml) in the presence of costimulating antibodies anti-CD28/CD49d (1μg/ml) (BD Biosciences). Cells with no antigen stimulation were included as negative controls. Mtb peptide megapool represents immunodominant epitopes from 90 Mtb antigens recognized by HLA class II-restricted CD4 T cells in diverse populations (35, 36). After 2h, GolgiStop and GolgiPlug (BD Biosciences) were added (0.5uL/200uL) to each well, and incubated for an additional 18h.

### PBMC staining and flow cytometry

After stimulation, cells were washed, stained with Live/Dead viability dye 1:1000 (Invitrogen), washed then surface antibody cocktail (αCD3 BV510 1:80; αCD8 BV570 1:80; αCCR7 BV785 1:80; αCCR6 FITC 1:80; αCXCR3 BV605 1:40; αCD161 APC-Fire750 1:80; αCD69 BV650 1:20; αCD154 BV711 1:80; and αVα7.2 BV421 1:40; (all BioLegend), αCD4 BUV496 1:80; αCD45RA BUV395 1:80; αCD25 BUV563 1:80; αCD39 BUV737 1:80; and αCD26 BUV805 1:80; (all BD Biosciences), αCD153 Alexa Fluor 488 1:20; (R & D Systems) diluted in Brilliant Violet buffer (BD Biosciences) was added then kept in the dark for 20 min at room temperature. Next, cells were fixed and permeabilized using eBioscience FOXP3/Transcription Factor kit (Invitrogen) for 30 minutes on ice. Cells were washed twice with 1X eBioscience diluent, then resuspended in intracellular antibody mix (αRORγT Alexa Fluor 647 1:40; αIFNγ BB700 1:160; (both BD Biosciences), αIL 17 PE 1:20; αT-bet PE-Dazzle 594 1:40; (both BioLegend), αKi-67 eFluor450 1:40; and αFoxP3 PE-Cy5.5 1:40; (both Thermo Fisher Scientific) in eBiosciences perm diluent for 20 minutes at room temperature in the dark. Next, cells were washed twice and fixed in 2% PFA. Data was acquired on Aurora Spectral Flow cytometer (Cytek Biosciences).

### Data and statistical analysis

Spectral fcs files were analyzed using SpectroFlo v3.0 (Cytek Biosciences) for unmixing and autofluorescence correction and T-cell subsets were identified using Flowjo v10 (Flowjo LLC). Antigen-responsive cytokine levels are reported after subtracting no antigen stimulation tests for each participant.

Statistical analysis was performed using SAS version 9.4 (SAS Institute Inc.). Chi-square or Fisher’s exact tests were used to compare categorical variables, and non-parametric tests were used to compare median values in study groups (Mann-Whitney U or Wilcoxon rank-sum tests) with Dunn’s *post-hoc* correction. To evaluate the independent contribution of T2D to different CD4 T cell subsets among TB patients, we performed GENMOD analysis to fit a generalized linear model to the data, using maximum likelihood estimation of the parameter vector β adjusted for sex, body mass index, and age. P-values were considered significant if ≤ 0.05 while p-values between 0.05 – 0.099 were considered borderline significant. Graphs were created using GraphPad Prism v9.0 (GraphPad Software).

## Results

### Study population characteristics

We selected 23 newly diagnosed TB patients with T2D (n=12) or without T2D (n=11) matched by age and sex (**Table 1**). Other sociodemographics were similar. 64% of TB-only patients had pre-T2D while TB-T2D patients had known their T2D diagnosis for a median of 8.8 years before the current TB episode. 11/12 of the T2D patients reported taking a glucose-lowering medication in the past month, mainly metformin (83%) (**Supplementary Table S1**). The participants with TB-T2D had significantly higher measures of glucose or estimates of insulin resistance and lower estimates of % of beta-cell function (**Table 1**). Complete blood counts were comparable between study groups except for a lower neutrophil: lymphocyte ratio in TB-T2D vs TB-no T2D (**Supplementary Table S2**). All patients without T2D had taken antimycobacterial drugs for 1 to 10 days, compared to 5 (42%) with TB-T2D (p=0.005). Other TB symptoms were similar by T2D status (**Supplementary Table S3**).

**Table 1:** Characteristics of Hispanic TB patients by type 2 diabetes status ^1^.

Characteristics	n	TB-No T2D	n	TB-T2D	p value^2^
Sociodemographic parameters					
**Age**, years (IQR)	11	45 (16.5)	12	44 (17)	1.000
**Male sex**	11	6 (55%)	12	7 (58%)	0.855
**Education up to Middle School**	11	7 (64%)	12	9 (75%)	0.890
**Smoking:**	11		12		0.217
Current smoker		2 (18%)		0 (0)	
Never or past		9 (82%)		12 (100%)	
Smoking pack-year index (IQR) ^3^	11	0 (21)	12	0 (2.4)	0.601
**BCG vaccine**	11	10 (91%)	12	10 (83%)	1.000
Obesity and central obesity					
**BMI underweight/normal**	11	9 (82%)	12	8 (67%)	0.640
**Waist-hip ratio - Females**	5	0.9 (0.04)	5	0.9 (0.01)	0.531
**Waist-hip ratio - Males**	5	0.9 (0.1)	6	0.95 (0.1)	**0.045**
T2D classification, history and laboratories					
***T2D and pre-diabetes classification***	11		12		**<0.001**
No T2D		4 (36%)		0 (0)	
Pre-T2D		7 (64%)		0 (0)	
T2D		0 (0)		12 (100%)	
**Years with T2D**	0	0 (0)	12	8.8 (8.2)	**<0.001**
**Glucose control and related biomarkers** ^4^					
Fasting glucose (mg/dl)	11	102 (23)	12	227.5 (139.5)	**<0.001**
Hemoglobin A1c (HbA1c) (%)	11	5.9 (0.5)	12	10.7 (2.8)	**<0.001**
Insulin resistance estimate (HOMA-IR)	10	2.1 (1.8)	11	3.3 (3.5)	**0.007**
% beta-cell function estimate (HOMA2)	10	66.9 (65.8)	11	13.8 (18.6)	**0.002**
Insulin (mU/L)	11	8.9 (6.3)	12	6.7 (5.5)	0.498
Vascular diseases					
Any macro/microvascular disease ^5^	11	3 (27%)	12	9 (75%)	**0.039**
Any macrovascular disease	11	1 (9%)	12	3 (25%)	0.590
Any microvascular disease	11	0 (0)	12	4 (33%)	0.093
Tuberculosis characteristics					
**Positive acid-fast smear**	11	11 (100%)	12	10 (83.33%)	0.478
**Days of TB treatment**	11		12		**0.008**
None		0 (0)		7 (58%)	**0.005**
1-10 days		11 (100%)		5 (42%)	

^1^ Data expressed as n (column %) for categorical or median (interquartile range) for continuous variables; ^2^ Chi-square for categorical variables with Yates correction and Wilcoxon rank sum test for continuous variables; ^3^ Cigarettes/day * # years)/20 cigarettes per pack; ^4^ Data from participants who did not have a fasting glucose measure or are taking insulin are excluded from reports for the corresponding glucose-related measures; ^5^ Reported diseases listed in the Methods.

BCG, bacille Calmette-Guerin; T2D, Type 2 diabetes; Pre-T2D, pre-type 2 diabetes; HOMA, homeostasis model assessment.

Bold values indicate significant differences with p value of 0.05 - 0.099 considered borderline significant.

### T2D is associated with a trend toward increased circulating Th1 and regulatory T cells

**Figure 1:**
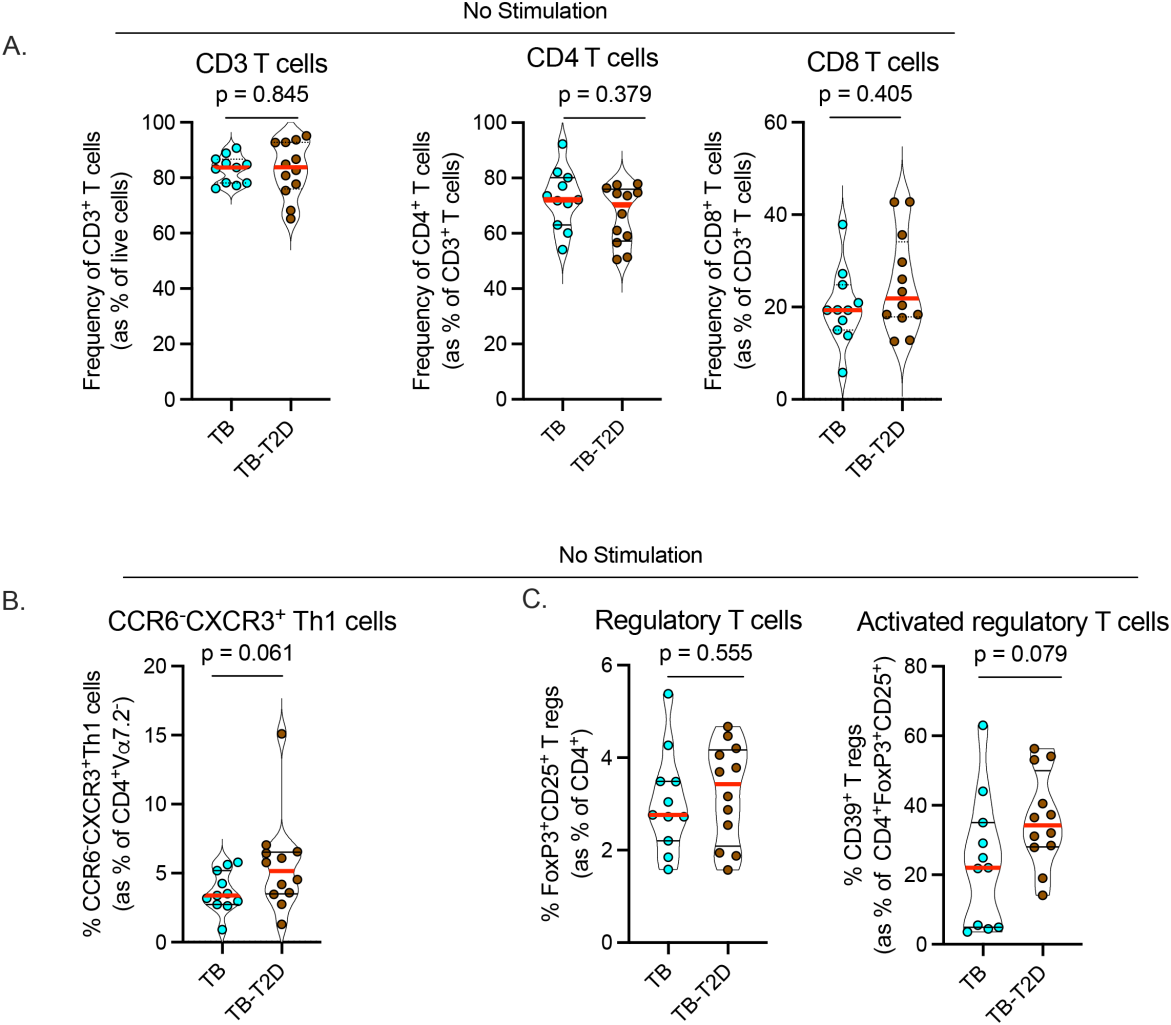
T2D is associated with alterations in Th1 and regulatory T cell differentiation states. We analyzed cryopreserved peripheral blood mononuclear cells (PBMC) from TB patients with or without T2D without antigen stimulation. After antibody staining, data were acquired by spectral flow cytometry. **(A)** Frequency of circulating CD3, CD4, and CD8 T cells between TB-T2D and TB no-T2D. **(B)** Marginal increase in circulating Th1 cells (CD4^+^Vα7.2^-^CCR6^-^CXCR3^+^) in TB-T2D. **(C)** Similar frequency of regulatory T cells (CD4^+^FoxP3^+^CD25^+^) (left) in TB-T2D and TB-noT2D and a marginal increase in activated regulatory T cells (regulatory T cells expressing CD39; CD4^+^FoxP3^+^CD25^+^CD39^+^) in TB-T2D. Statistics: Wilcoxon rank-sum test, p= 0.05 – 0.099 considered borderline significant.

To identify alterations in circulating T cells in people with TB by T2D status, we designed a high-parameter spectral flow cytometry panel to measure T cell maturation states, proliferation, differentiation, and effector functions (**Supplementary Figure 1**). We found no difference in the frequency of circulating bulk CD3, CD4, and CD8 T cells (**Figure 1A**). Further analysis was focused on the characterization of circulating CD4 T cells since this broad subset plays a prominent role in TB control (11, 12, 15, 37). First, we evaluated cells without antigen stimulation and found a significantly higher frequency of CD4 T cells expressing the Th1 master transcription factor T-bet in TB-T2D compared to TB alone. Accordingly, T2D patients had a borderline higher proportion of circulating Th1 T cells (CD4^+^Vα7.2^-^CCR6^-^CXCR3^+^; **Table 2**, **Figure 1B**). For regulatory T cells, there was no difference in the frequency of their master transcription factor (FoxP3), nor their cell surface markers (FoxP3^+^CD25^+^). However, activated (CD39^+^) regulatory T cells were borderline higher in T2D (**Figure 1C**, **Table 2**). Together, these findings suggest that in TB-T2D patients versus TB alone, there are no differences in the overall frequencies of CD4 and CD8 T cells, but within the CD4 T cell subsets, there are increases of Th1 and activated regulatory T cells.

**Table 2:** Proportion of CD4 T cell subsets among TB patients, by type 2 diabetes status^1^.

CD4 T cell category and subset (%)	TB-No T2D (n=11)	TB-T2D (n=12)	P value^2^
No Stimulation CD4 T cell phenotype			
**Th1 lineage and differentiation**			
Th1 transcription factor (T-bet+)	17 (12)	29.7 (15.1)	**0.039**
Th1 (CD4+Vα7.2-CCR6-CXCR3+)	3.4 (2.5)	5.2 (3.0)	**0.061**
**T regulatory lineage and differentiation**			
Regulatory T cell transcription factor (FoxP3+)	3.6 (2.1)	3.7 (2.0)	0.538
T regulatory (CD4+FoxP3+CD25+)	2.7 (1.3)	3.4 (1.9)	0.559
T regulatory, activated (CD4+FoxP3+CD25+CD39+)	22 (30)	34.3 (18.7)	**0.079**
**Th17 lineage and differentiation**			
Th17 transcription factor (RORγt+)	5.4 (5.9)	4.4 (5.3)	0.712
Th17, Subset 1 (CD4+Vα7.2-CD26+CD161+)	7.7 (7.8)	15.9 (6.2)	**0.006**
Th17, Subset 2 (CD4+Vα7.2-CCR6+CXCR3-)	14.4 (17.2)	30.8 (12.6)	**0.015**
Th1* (Th1/Th17; CD4+Vα7.2-CCR6+CXCR3+)	3 (3.9)	6.7 (3.4)	**0.001**
**CD4 T cell maturation state^2^**			
Naive	49.8 (34.6)	32.5 (22.5)	**0.013**
Central memory	16.1 (23.5)	33.8 (11.2)	**0.053**
Effector memory	15.1 (9.7)	15.2 (13.7)	0.424
Terminally differentiated effector memory	4.9 (10.8)	3.7 (11.7)	0.601
CD4 T cell phenotype after Mtb300 stimulation			
**Proliferation by maturation state**			
Naive, Ki67+	0.4 (0.9)	0.2 (0.6)	0.268
Central memory, Ki67+	1.5 (0.9)	0.6 (0.2)	**0.006**
Effector memory, Ki67+	2.2 (1.0)	1.4 (0.9)	**0.019**
Terminally differentiated effector memory, Ki67+	1.8 (1.5)	1.1 (1.0)	0.372
**Activation phenotype of CD4+ cells**			
CD69+CD153+	0.4 (0.5)	0.6 (0.7)	0.331
CD154+CD69+	0.3 (0.4)	0.5 (0.2)	**0.045**
CD154+CD153+	0.1 (0.1)	0.2 (0.2)	0.116
**Cytokine responses of CD4+ cells**			
IFNγ+	0.2 (0.2)	0.2 (0.2)	1.000
IL17+	0.2 (0.6)	0.2 (0.2)	0.538
TNFα+	0.7 (0.7)	0.5 (1.1)	0.782
TNFα+IFNγ+	0.1 (0.1)	0.1 (0.1)	0.806
IFNγ+IL17+	0.0 (0.01)	0.0 (0.0)	0.504
TNFα+IL17+	0.0 (0.0)	0.0 (0.0)	0.854

^1^Data shown as median (interquartile range) of the % of each CD4 T cell subpopulation when compared to all CD4 T lymphocytes as denominator; ^2^Wilcoxon rank sum test. ^2^: Naïve (CD45RA+CCR7+); Central memory (CD45RA-CCR7+); Effector memory (CD45RA-CCR7-); Terminally differentiated effector memory (CD45RA+CCR7-).

MTB300, *Mycobacterium tuberculosis* antigen megapool of 300 peptides from 90 Mtb antigens.

Bold values indicate significant differences with p value of 0.05 - 0.099 considered borderline significant.

### T2D is associated with an increase in circulating Th17 cell subsets in TB

**Figure 2:**
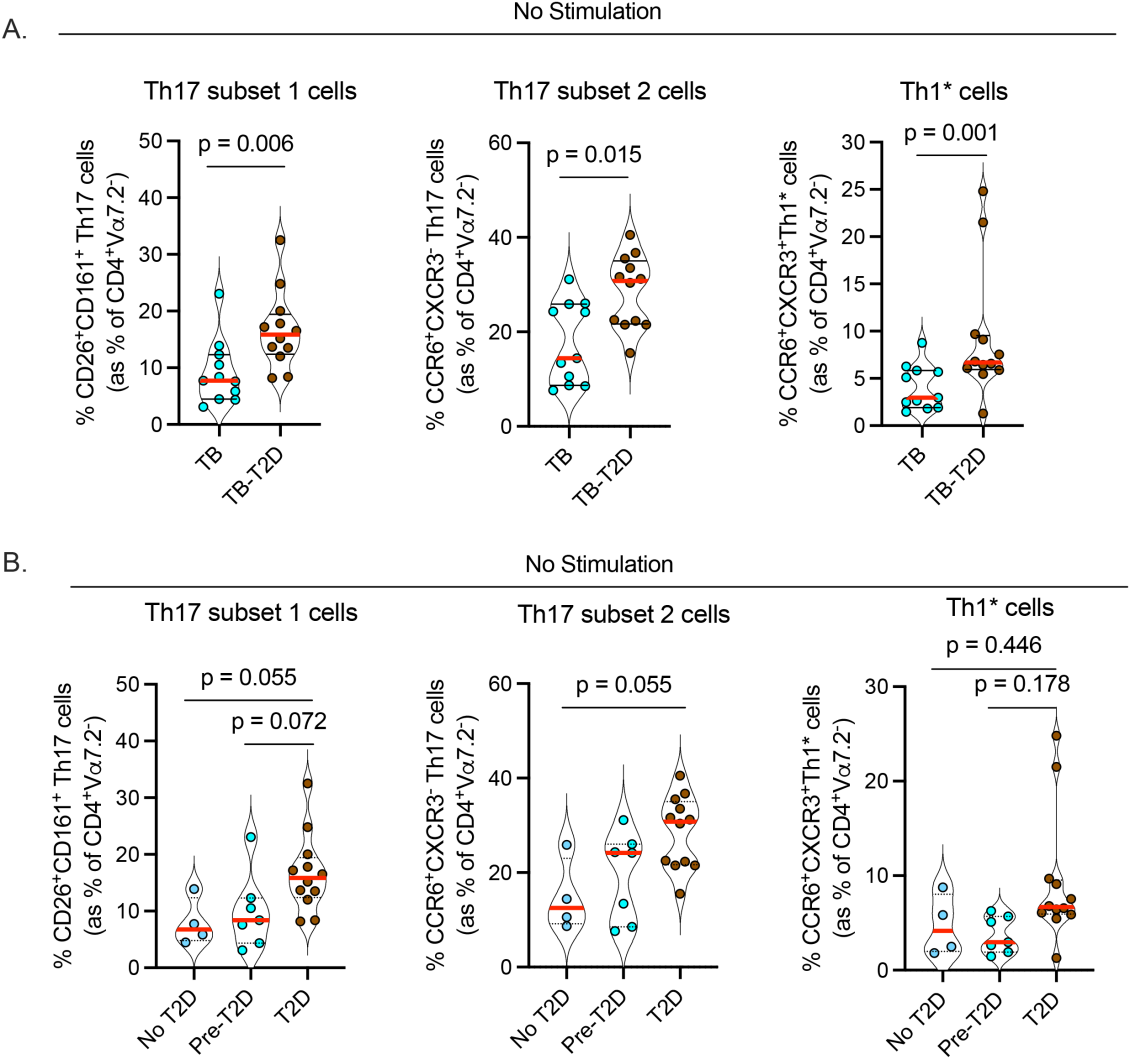
T2D is associated with an increase in circulating Th17 cell subsets in TB. Cryopreserved PBMC were processed as in **Figure 1** without antigen stimulation followed by characterization of Th17 cells of CD4^+^Va7.2^-^ helper T cells. **(A)** Increased frequencies of circulating Th17 cells; subsets 1 (CD4^+^Vα7.2^-^CD26^+^CD161^+^); subset 2 (CD4^+^Vα7.2^-^CCR6^+^CXCR3^-^) and Th1* (Th1/Th17: CD4^+^Vα7.2^-^CCR6^+^CXCR3^+^) in TB-T2D. **(B)** Frequency of Th17 cell subsets stratified by T2D status; Statistics: Wilcoxon rank-sum test, p= 0.05 – 0.099 considered borderline significant.

Emerging evidence suggests a beneficial role for Th17 and Th17-like cells in controlling Mtb in humans (23–25) and animal models (38, 39). To identify Th17 cell subsets, we used the gating strategy we described previously (29) that defines Th17 cells based on the expression of cell surface markers (CD26 and CD161) and chemokine receptors (CCR6 and CXCR3) (40, 41) among CD4 T cells (see extended data of 1xN plots (**Supplementary Data Sheet 1**)). We excluded mucosa-associated invariant T cells (MAIT) that also express CD26 and CD161 in addition to the canonical MAIT cell marker Vα7.2 (42). We identified three categories of Th17 cells (29); subset 1 (CD4^+^Vα7.2^-^CD26^+^CD161^+^), subset 2 (CD4^+^Vα7.2^-^CCR6^+^CXCR3^-^), and Th1* (Th1/Th17), (CD4^+^Vα7.2^-^CCR6^+^CXCR3^+^). We found a significant increase in circulating subset 1, subset 2, and Th1* cells in TB-T2D versus TB (**Figure 2A**, **Table 2**), despite finding no difference in the expression of RoRγt, the master regulatory transcription factor for the Th17 lineage (**Table 2**). We further stratified participants into no T2D, pre-T2D, and T2D to screen for the extent of dysglycemia when Th17 phenotypes change. Results suggested a gradual increase in the Th17 phenotypes through the No T2D < pre-T2D < T2D states, but significant increases were only detected for Th17 subsets 1 and 2 (**Figure 2B**). Characterization of the expression of CD26, CD161, and CCR6 on CD8^+^Vα7.2^-^ T cells also revealed an increase in all three circulating cytotoxic type 17 cell subsets in TB-T2D compared to TB-only (**Supplementary Figure 2a**). Thus, T2D is associated with an increase in three subsets of circulating Th17 cells in people with TB, and this shift may start at a pre-T2D stage.

### T2D is associated with altered CD4 maturation states in TB

**Figure 3:**
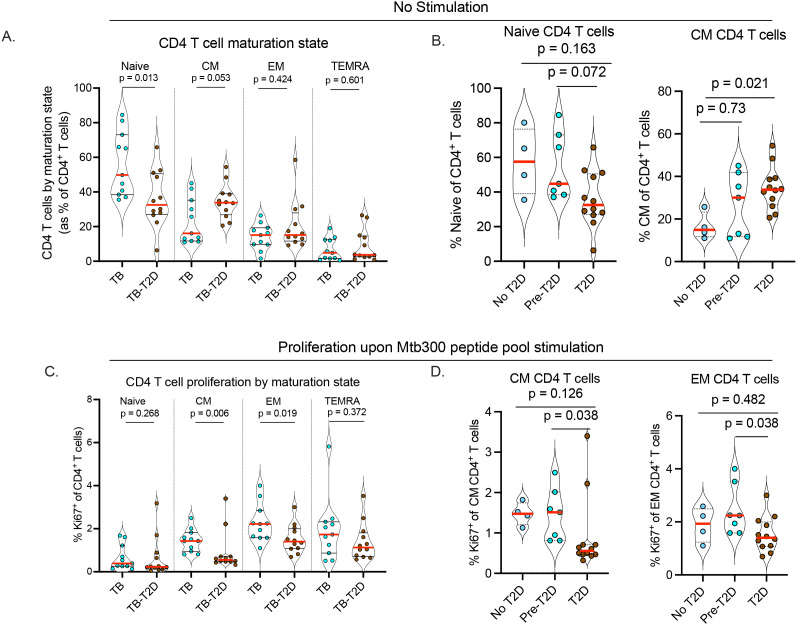
T2D is associated with altered CD4 T cell maturation state and reduced proliferation of Mtb-antigen-responsive memory CD4 T cells. To determine the T cell maturation state, cryopreserved PBMC were thawed, rested, and counted as described in **Figure 1**. **(A)** Alteration of CD4 T cell maturation state in TB-T2D and TB-noT2D as defined by the expression of CD45RA and CCR7 as Naive: CD45RA^+^CCR7^+^; Central Memory (CM): CD45RA^-^CCR7^+^; Effector Memory (EM): CD45RA^-^CCR7^-^; Terminally differentiated effector (TEMRA): CD45RA^+^CCR7^-^. **(B)** The frequency of Naïve and central memory CD4 T cells stratified by T2D status. For the proliferation of Mtb-antigen-responsive CD4 T cells, 1x10^6^ live cells were stimulated with Mtb300 megapool antigen (2μg/ml) for a total of 20 hours in the presence of costimulatory antibodies anti-CD28 and anti-CD49d with Golgi Stop and Golgi Plug added 2 hours after the start of stimulation. CD4 T cell proliferation was determined by intracellular staining for the proliferation marker Ki67, and cells were analyzed by spectral flow cytometry. **(C)** The proportion of Ki67 expressing CD4 T cells was determined based on their maturation state defined as naive, central memory, effector memory, and terminally differentiated effector cells. **(D)** The proliferation of Mtb-antigen responsive central and effector memory CD4 T cells stratified by T2D status; Statistics: Wilcoxon rank-sum test, p= 0.05 – 0.099 considered borderline significant.

To further characterize CD4 T cells during TB and with or without T2D, we analyzed their maturation states based on CCR7 and CD45RA expression, with classification into naïve (CCR7^+^CD45RA^+^), central memory (CM: CCR7^+^CD45RA^-^), effector memory (EM: CCR7^-^CD45RA^-^), and terminally differentiated memory (TEMRA: CCR7^-^CD45RA^+^) T cells (25, 26, 29). The frequency of circulating naïve CD4 T cells was significantly lower in TB-T2D than in those with TB alone (**Figure 3A**, **Table 2**). This difference was accompanied by a trend towards more circulating mature CD4 T cells, particularly central memory cells (**Figure 3A**, **Table 2**). Stratified by the extent of dysglycemia, the results suggested that as T2D was developing, the frequency of circulating naïve CD4 T cells was decreased while those with a central memory phenotype were increased (**Figure 3B**). We found no difference in CD8 T cell maturation states, except for a trend toward higher circulating memory CD8 T cells in TB-T2D than in TB alone (**Supplementary Figure 2b**). Together, T2D is associated with a shift from fewer naïve to more mature circulating CD4 T cell states.

### T2D is associated with lower proliferation of Mtb antigen-responsive CD4 T cells according to their maturation state

To assess the capacity of T cells to proliferate in response to stimulation, we measured the intracellular expression of the proliferation marker, Ki67, on CD4 T cells after 20-hour stimulation with the Mtb300 antigen megapool. We found no difference in the proportion of Ki67^+^ cells in the bulk CD4 T cell population in response to antigen stimulation between TB-only and TB-T2D (data not shown). Given the differences by T2D status in CD4 T cell maturation states (**Figure 3A**), we evaluated whether Ki67 expression differed in Mtb300-stimulated CD4 T cells by maturation state. We found no difference in the proliferation of antigen-stimulated naïve CD4 T cells, but the proliferation of central memory and effector memory CD4 T cells was significantly reduced in TB-T2D compared to TB (**Figure 3C**). Further stratification of participants by extent of dysglycemia showed a lower frequency of Ki67^+^ T cells among the central memory or effector memory CD4 T cells in T2D, and not in the pre-T2D stage (**Figure 3D**). In summary, T2D is associated with decreased proliferation of central and effector memory CD4 T cells in people with TB.

### Increased frequencies of activated (CD69^+^CD154^+^) CD4 T cells in response to Mtb antigens in T2D-TB patients compared to TB alone

To compare the effector responses of CD4 T cells to Mtb antigens in TB by T2D status, we stimulated PBMC with the Mtb300 antigen megapool and measured the expression of the activation markers CD69 and CD154 in combination with CD153. While we found no difference by T2D status in the expression of CD69, CD153, or CD154 as single markers (data not shown), the frequency of CD69^+^CD154^+^ T cells was higher in TB-T2D than in TB alone (**Table 2**). We also measured the frequency of CD4 T cells expressing IFNγ, IL17, and TNFα after Mtb300 stimulation. We found no differences in the frequencies of any of the three cytokines individually or as combinations on bulk CD4 T cells between TB-T2D and TB-only individuals (**Table 2**). When we combined the groups and assessed IL17 production by Th17 cell subsets 1 and 2, we found that Th17 subset 1 preferentially produced IL17 compared to Th17 subset 2 in response to Mtb300 peptide pool stimulation (**Supplementary Figure 3**), in concurrence with our previous finding (29). Taken together, our analysis has revealed that T2D differentially alters distinct features of circulating T cells in people with TB, including maturation, proliferation, differentiation, and the activation of Mtb antigen-responsive CD4 T cells, despite similar cytokine responses of Mtb-responsive CD4 T cells in TB patients with or without T2D.

### Independent contribution of T2D to alterations in CD4 T cells

To determine the independent contribution of T2D to the CD4 T cell characteristics with significant or borderline significant associations by univariable analysis (**Figures 1**-**3**, **Table 2**), we performed a multivariable analysis with variable selection guided by host characteristics that differed by T2D status and were unrelated to glucose control (**Table 1**). Potential confounders included sex, vascular diseases, and days of TB treatment. The estimated beta coefficients and adjusted p-values are shown in **Table 3**. Among CD4 T cells without antigen stimulation, individuals with T2D had a higher proportion of cells committed to Th1 lineage (T-bet+), or Th1 and Th17 (subsets 1, 2, and Th1*), and activated regulatory T cell subsets. T2D also remained independently associated with a shift in maturation states with lower proportions of naïve T cells and higher central memory T cells. When PBMC were stimulated with the Mtb300 antigen megapool, T2D remained associated with an increase in the proportion of CD69^+^CD154^+^ activated T cells, but differences in proliferation were no longer significant.

**Table 3:** Independent contribution of type 2 diabetes to alterations in different CD4 T-helper cell subsets among TB patients, by multivariable analysis ^1^.

CD4 T cell subset category and subset	Adj β coef (95% CI) T2D/No T2D	Adjusted p value
No Stimulation CD4 T cell phenotype		
**Transcription factors by CD4 T cell lineage**		
T-bet+ (Th1 cell)	10.12 (1.42,18.83)	**0.023**
**CD4 T cell differentiation phenotypes**		
Th1 (CD4+Vα7.2-CCR6-CXCR3+)	3.03 (0.45,5.62)	**0.022**
Th17, Subset 1 (CD4+Vα7.2-CD26+CD161+)	8.16 (8.16,3.28)	**0.013**
Th17, Subset 2 (CD4+Vα7.2-CCR6+CXCR3-)	10.57 (2.84,18.3)	**0.007**
Th1* (Th1/Th17; CD4+Vα7.2-CCR6+CXCR3+)	5.77 (0.6,10.94)	**0.029**
T regulatory, activated (CD4+FoxP3+CD25+CD39+)	1.07 (-0.14,2.28)	**0.084**
**CD4 T cell maturation state**		
Naive	-14.97 (-31.21,1.27)	**0.071**
Central memory	11.15 (-0.7,22.38)	**0.051**
CD4 T cell phenotype after Mtb300 stimulation		
**Proliferation by maturation state**		
Central memory, Ki67+	-0.21 (-0.95,0.53)	0.570
Effector memory, Ki67+	-0.3 (-0.96,0.36)	0.374
**Activation phenotype in CD4+ cells**		
CD154+CD69+	0.21 (0.007,0.41)	**0.042**

^1^GENMOD test statistic used to estimate adjusted beta coefficients (adj β coef) of TB patients with T2D versus no T2D, controlling for sex, days of TB treatment, and reported vascular complications. CI, Confidence intervals.

Bold values indicate significant differences with p value of 0.05 - 0.099 considered borderline significant.

We performed additional analyses focusing on the association between Th17 cell subsets and markers of TB severity. Our findings indicate an association between Th17 cell subsets and T2D comorbidity in TB patients. Since T2D has been associated with adverse TB outcomes (27, 43–47), we examined whether a higher proportion of Th17 cells could be a contributor, by evaluating associations with features of a more severe TB. **Table 4** identified associations, after controlling for T2D (already known to have higher Th17 cells), as well as TB treatment initiation within 1–10 days of enrollment and age (associations by univariable analysis). Sex was not associated, and due to the small sample size, not controlled for in the multivariable models. We found correlations between the Th17 subset 1 and blood inflammatory biomarkers (platelets, neutrophils, and C-reactive protein) and duration of reported TB symptoms. Th17 subset 2 was associated with high blood pressure, higher platelet counts, and smear grade, and the Th1* subset was also associated with a higher smear grade. Together, these data support the independent contribution of T2D to alterations in CD4 T cell phenotypes in TB patients and suggest likely contribution of Th17 cells to poor outcomes of TB.

**Table 4:** Multivariable models on associations between the proportion of each Th17 subset and characteristics of the host, including measures of TB severity^1^.

	Th17 subset 1		Th17 subset 2		Th1*	
	β estimate	P value	β estimate	P value	β estimate	P value
Models in all participants, controlling for T2D, initiation of TB treatment and age at 44 y cut-off						
**Sociodemographics**						
Age ≥44 y (18–43 y)	-4.197	0.077	0.761	0.816	-4.941	0.006
Sex (Female)	0.297	0.903	-1.128	0.737	2.399	0.173
**Comorbidities**						
Type 2 diabetes	7.123	**0.020**	0.010	**0.010**	7.456	**0.001**
High blood pressure	4.900	0.104	9.021	**0.023**	3.187	0.165
**Inflammatory markers**						
Platelets	0.025	**0.004**	0.028	**0.027**	0.003	0.766
Neutrophils	1.236	**0.010**	0.437	0.554	0.515	0.245
C-reactive protein	0.042	**<0.0001**	0.020	0.121	0.011	0.139
**Duration of TB symptoms**						
Chest pain duration	0.056	**0.003**	0.034	0.259	-0.007	0.707
Fever/chills	0.056	**0.013**	0.057	0.089	-0.011	0.586
Productive cough	0.042	0.097	0.002	0.963	-0.012	0.541
**Smear score**	2.423	**0.041**	1.005	0.569	2.814	**<0.0001**
Neg	REF		REF		REF	
1+	-1.008	0.813	-2.453	0.638	-0.165	0.953
2+	6.987	0.140	14.139	0.015	5.913	0.056
3+	5.669	0.181	2.591	0.616	6.865	0.013
Models in participants with T2D, controlling for HbA1c, TB treatment initiation and age at 44 y cut-off						
**Meds in last month^2^**						
Metformin	11.998	**0.008**	4.404	0.507	-1.291	0.800
Sulphonylurea	4.267	0.261	0.978	0.836	9.686	**<0.0001**

**^1^** All models are adjusted for type 2 diabetes, days of TB treatment, and age (44 y cut-off); ^2^Some T2D patients took both medications in the past month.

Bold values indicate significant differences.

## Discussion

We studied samples from well-defined Mexican-Hispanic TB patients with or without T2D (48) to characterize circulating CD4 T cells using an extensive T cell antibody panel (29) and spectral flow cytometry. We found alterations by TB-T2D status in the maturation and differentiation state of unstimulated CD4 T cells and in responses to Mtb antigen stimulation. TB patients with T2D had increased circulating Th17 and Th1 cells, and their maturation state was skewed from naïve to central memory CD4 T cells. Upon stimulation, the Mtb antigen-responsive memory CD4 T cells in TB-T2D (versus TB alone) had a highly activated Mtb antigen-responsive phenotype (CD69^+^CD154^+^).

Elucidating alterations due to T2D in TB patients, and their impact on disease progression has been challenging, at least partly due to discrepancies between studies. Previous studies have reported CD4 T cell differentiation patterns (Th1, Th17, T regulatory) unique to TB-T2D patients based on cytokine secretion (19–21). However, assay conditions can influence cytokine responses, including the antigen’s nature and the sample type. Moreover, focusing only on cytokines may overlook essential CD4 T cell features, such as inhibitory receptor expression and maturation state, which influence cytokine production (49, 50). Additionally, plasma cytokine levels are unreliable indicators of CD4 T cell activity, as multiple cell types produce the same cytokines (51). We took a different approach by identifying T2D-associated alterations of CD4 T cell populations *ex vivo* without antigen stimulation and therefore not influenced by assay conditions. Using this cytokine-agnostic approach, our findings extend the current understanding of the specific differences in CD4 T cells in T2D patients with TB.

Our results agree with previous studies suggesting an increase in pro-inflammatory Th1 cells in TB-T2D (19, 20). We found a significantly higher proportion of unstimulated cells expressing transcription factor T-bet (master regulator of Th1 cell development) and borderline higher proportion of CD4 T cells expressing CXCR3, a chemokine receptor associated with Th1 phenotype, even though Mtb antigen stimulation did not result in a higher proportion of cells expressing IFNγ and TNFα, in agreement with prior reports (21, 22). In other studies, a higher Th1 response has been associated with and attributed to reduced regulatory T cells and IL10 production (20, 21). In contrast, we found no difference in regulatory T cells by T2D status, but a trend towards higher frequency of activated regulatory T cells in TB-T2D. In addition to technical differences, variations between studies may be due to heterogeneity in study populations and our rigorous characterization and adjustment of host characteristics to identify the independent contribution of T2D.

Our results are consistent with previous studies showing the enhancement of Th17 cells (as previously defined by IL17 expression) in TB-T2D (20, 22). Here, we expand Th17 cell characterization beyond IL17 production. Th17 cell differentiation is regulated by several cytokines (IL6, IL23, and IL1-β) which collectively promote expression of the transcription factor RORγt, essential for Th17 lineage commitment (52, 53). While we found no difference in the expression of RORγt in CD4 T cells by T2D status, we found a significant increase in circulating Th17 cell subsets defined by cell surface markers and chemokine receptors (40, 41). More studies are required to identify the mechanisms that drive the increase in circulating Th17 subsets in TB-T2D. However, the increased Th17 cells could reflect an expansion of memory Th17 cells, which can respond to IL6, IL23, and IL1-β (that are elevated in T2D (19)) and amplify Th17 cell activity (54). Little is known about the Th17 cell subsets reported in our study, including whether they are distinct populations, transitional states, or both. We have demonstrated here, in agreement with our prior study (29), that Th17 cell subset 1 and subset 2 differ in the ability to produce IL17 in response to Mtb antigen stimulation (29). Thus, we find that in TB patients, T2D is associated with an expansion of Th17 cell subsets in circulation despite similar IL17 production in response to Mtb antigen stimulation. Our findings may appear paradoxical regarding Th17 cell control of TB since we found increases in Th17 cells in those with T2D-TB, whose outcomes as a group are poorer than in those without T2D (8, 10, 18, 55). The heterogeneity of Th17 cells is an important determinant of their role during infections, as Th17 cells can be both protective and pathologic (56–58). Previous studies have linked Th17 cell responses to severe disease in people with active TB (27, 43–47) in mechanisms that include both IL17-specific and cytokine-independent manner. The pathogenicity of Th17 cells has also been reported in multiple noninfectious immunological disorders where they can contribute to tissue/organ damage and dysfunction. Therefore, our results are consistent with the hypothesis that Th17 responses can also be pathogenic in the context of TB. Since the prevalence of cavities in TB patients is higher with TB-T2D (59), a future topic of study will be to determine whether the frequency and properties of Th17 cells are associated with, and contribute to the development of cavitary TB.

T cell maturation into memory states is crucial for controlling Mtb infection since memory T cells provide long-term immunity and respond rapidly to subsequent exposures (60). We found lower circulating naïve CD4 T cells with the accompanying trend toward increased central memory CD4 T cells in TB-T2D. Although not significant after multivariable comparison, we found that central and effector memory CD4 T cells had a lower proliferative potential upon Mtb antigen stimulation in TB-T2D than in TB-only. Future studies are needed to address the implication of T2D regulation of CD4 T cell maturation states and how that alters responses to Mtb antigens. The trend toward more mature circulating CD4 T cells may reflect a greater history of infectious exposures or persistent infections associated with the generation of memory T cells at the expense of naive T cells in TB-T2D. Additionally, a persistent increase in memory T cells may lead to pathologic inflammatory responses that worsen TB disease. Chronically activated memory T cells can exhibit aberrant cytokine production and homing capabilities (61, 62), both of which are important for controlling organ-specific diseases like TB.

Taken together, our findings suggest a specific proinflammatory environment in people with TB-T2D compared to those with TB-only (reduced naïve CD4 T cells, increased central memory and Mtb antigen-responsive CD4 T cells, and elevated Th1 and three Th17 cell subsets) that together may contribute to tissue damage that worsens TB outcomes. Our findings also suggest that Th17 cells may be contributing to TB disease severity, independent of T2D. Interestingly, differences in associations with features of TB disease severity or comorbidities suggest biological differences between the three Th17 subsets, although larger sample sizes will be important to confirm these variations. This pilot study is limited by its small sample size, particularly when stratified into no T2D, pre-T2D, and T2D. To increase the chance of the application of our results to other settings, we stringently matched our study participants by sex and age, and T2D participants had a median HbA1c of 10.7%. Thus, our findings provide insights into the relationships between T2D stage and CD4 T cell alterations. The second limitation of our study is that T2D medication use was self-reported, and we cannot confirm compliance. We also acknowledge that we did not assess the properties of the antigen-presenting cells that play a major role in determining CD4 T cell differentiation trajectory in the present study, and their role cannot be ruled out. Lastly, the observational nature of our study does not allow us to distinguish whether alterations in T cells explain higher TB risk or are a consequence of higher Mtb load in TB-T2D vs TB alone. Nonetheless, our study demonstrates that T2D is associated with distinct differences in CD4 T cell responses in people with TB, including lower circulating naïve cells, increased circulating Th17 cells, and higher activation of Mtb antigen-responsive CD4 T cells compared to those without T2D. These data suggest that TB-T2D patients are more likely to have deleterious outcomes like exacerbated lung damage due to an increase in Th17 cells (e.g. cavitary TB). We propose that further investigation of these altered CD4 T cell characteristics be evaluated in T2D patients at different stages of TB, and in complement with animal models, to identify pathways in Th17 cell development that favor the generation of protective Th17 cell subsets that can be targets of host-directed therapies for both TB, particularly in patients with T2D.

## Data Availability

The raw data supporting the conclusions of this article will be made available by the authors, without undue reservation.
