# Supplementary material for: Increased circulating Th17 cells and altered CD4 T cell maturation and differentiation in active tuberculosis with type 2 diabetes: a pilot study
Source: Front Immunol. 2025 Sep 9;16:1637868. doi: 10.3389/fimmu.2025.1637868 (PMC12454991; doi:10.3389/fimmu.2025.1637868)
Supplement: Supplementary file 1 [file DataSheet1.docx]

**Increased circulating Th17 cells and altered CD4 T cell maturation and differentiation in active tuberculosis with type 2 diabetes: a pilot study**

Paul Ogongo^1*^, Yoscelina E. Martinez-Lopez^2^, Anthony Tran^1^, Cecilia S. Lindestam Arlehamn^3,4^, Alessandro Sette3,5, Ilse A. Dominguez-Trejo2, Lizette Garza6, America M. Cruz-Gonzalez7, Raul Loera-Salazar8, Javier E. Rodríguez-Herrera8, Genesis P. Aguillón-Durán2,9, Esperanza M. Garcia- Oropesa9, Joel D. Ernst1, Blanca I. Restrepo 2,9,10

Extended Data: 1xN plots of Th17 cell markers

The goal of these 1XN plots is to show that there is an overlap in the expression of CD26, CD161, and CCR6 as phenotypic markers of Th17 cells. Prior studies had shown that individually CD26 (Bengsch et al, 2012), CD161 (Cosmi et al, 2008), and CCR6 (Singh et al, 2022) can identify IL17-producing CD4 T cells (Th17 cells), and that there is co-expression between these markers. However, given the heterogeneity of Th17 cells, a single marker is not sufficient to identify all the Th17 cells. The plots shown here are gated on total live cells in PBMC stimulated with Mtb300 peptide pool and therefore include non-CD4 T cells that express these markers. The plots also concur with prior studies that CD26^+^, CD161^+,^ or CCR6^+^ cells produce IL17 upon antigen stimulation (in our case, with Mtb antigen). In addition, all three markers express RoRγT (the master transcription factor for Th17 cells) at varying levels. Together with Supplementary figure 3 and our prior study (Ogongo et al, 2024), we show the functional heterogeneity of Th17 cells (with regards to IL17 production), with Th17 subset 1 particularly enriched for Mtb-specific IL17 production.

#
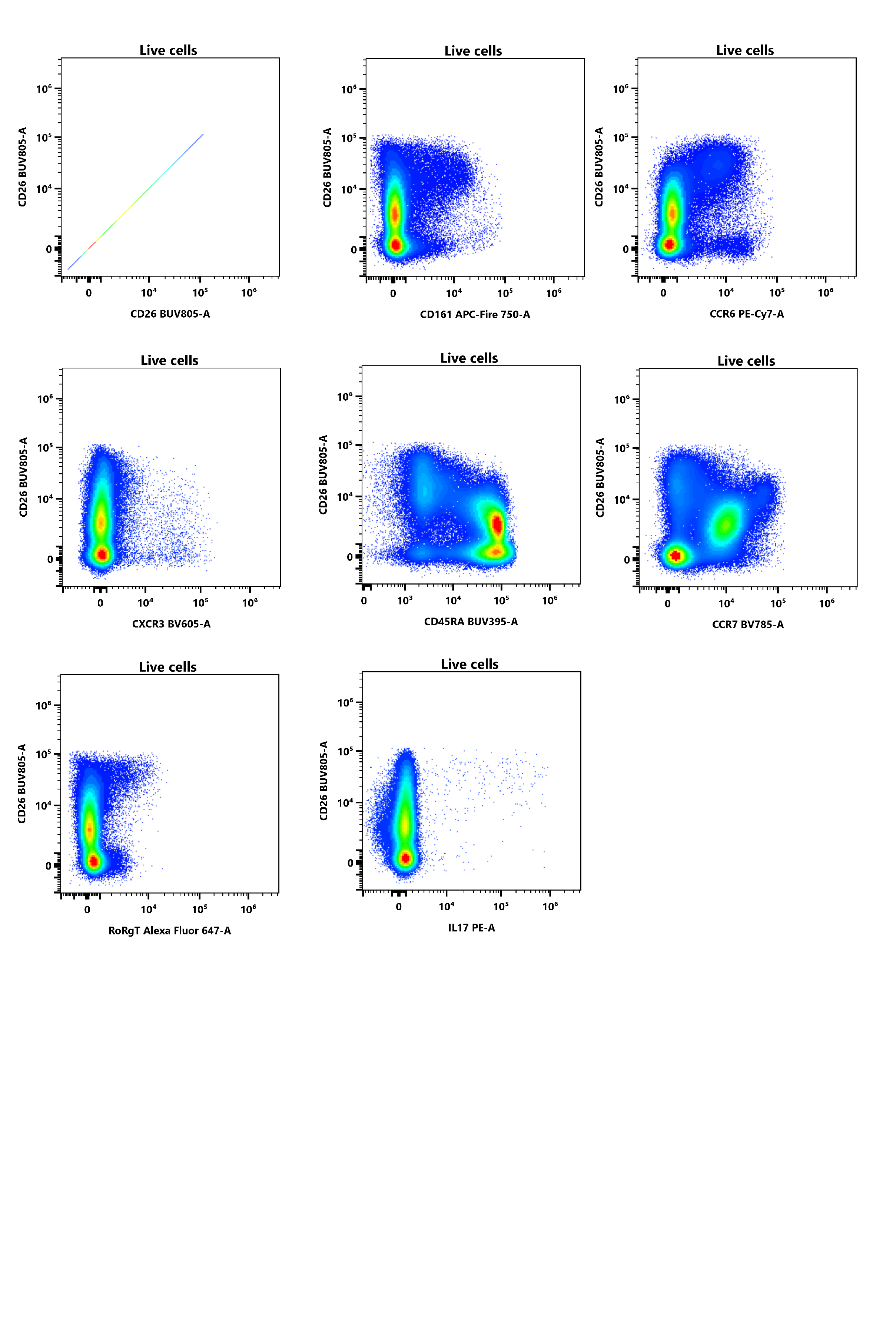
CD26

The plots show co-expression of CD26 with select T cell markers

#
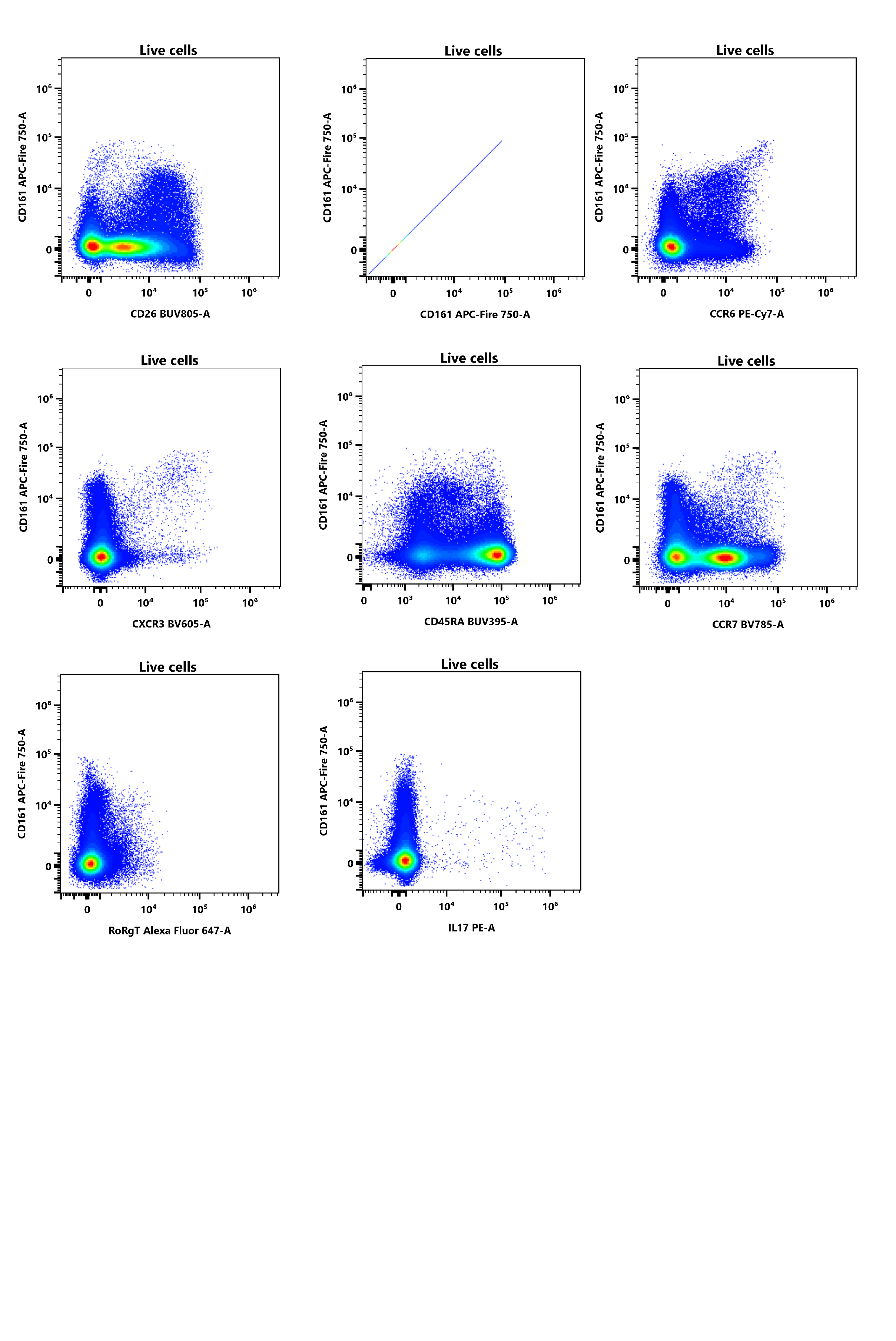
CD161

The plots show co-expression of CD161 with select T cell markers

#
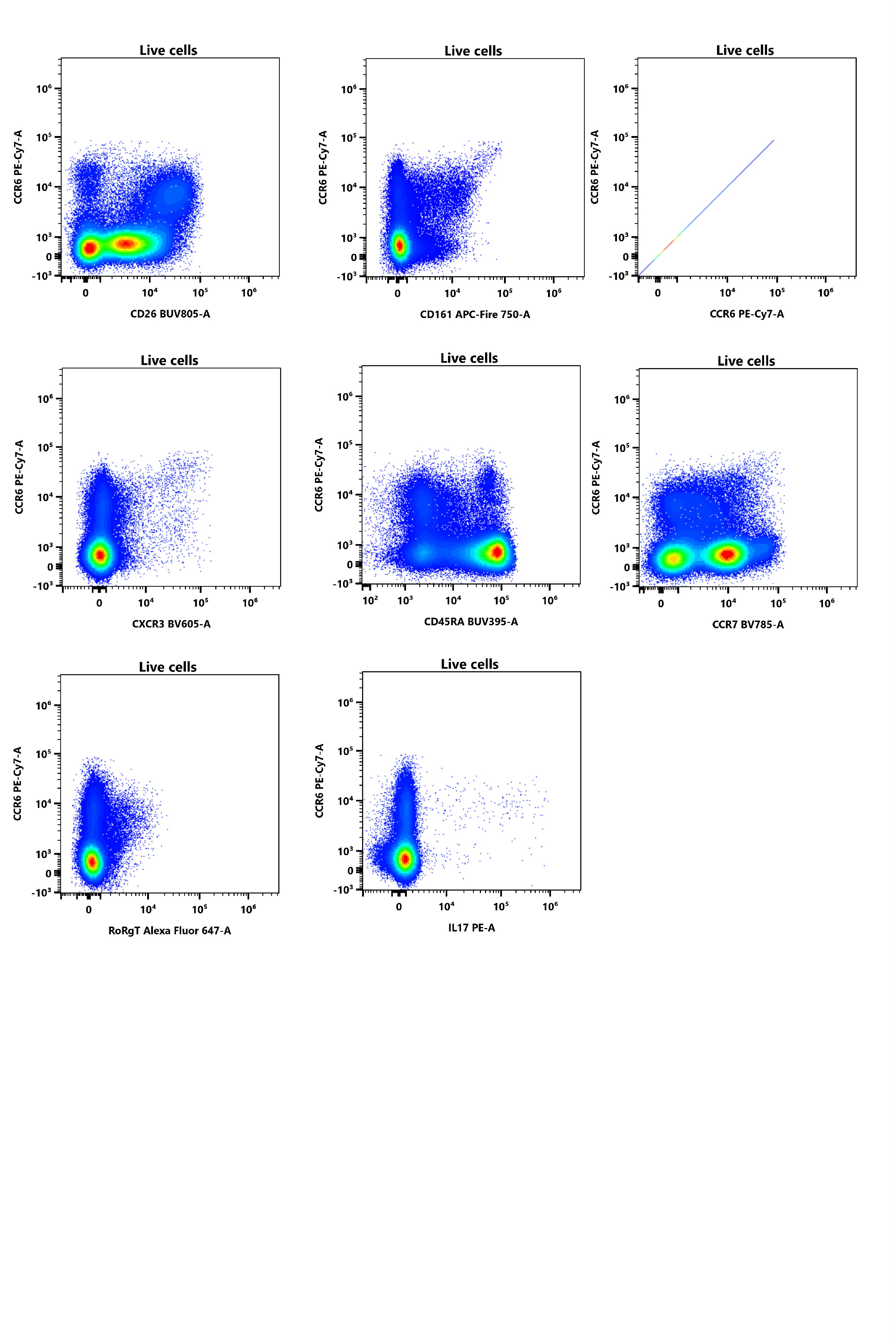
CCR6

The plots show co-expression of CCR6 with select T cell markers

#
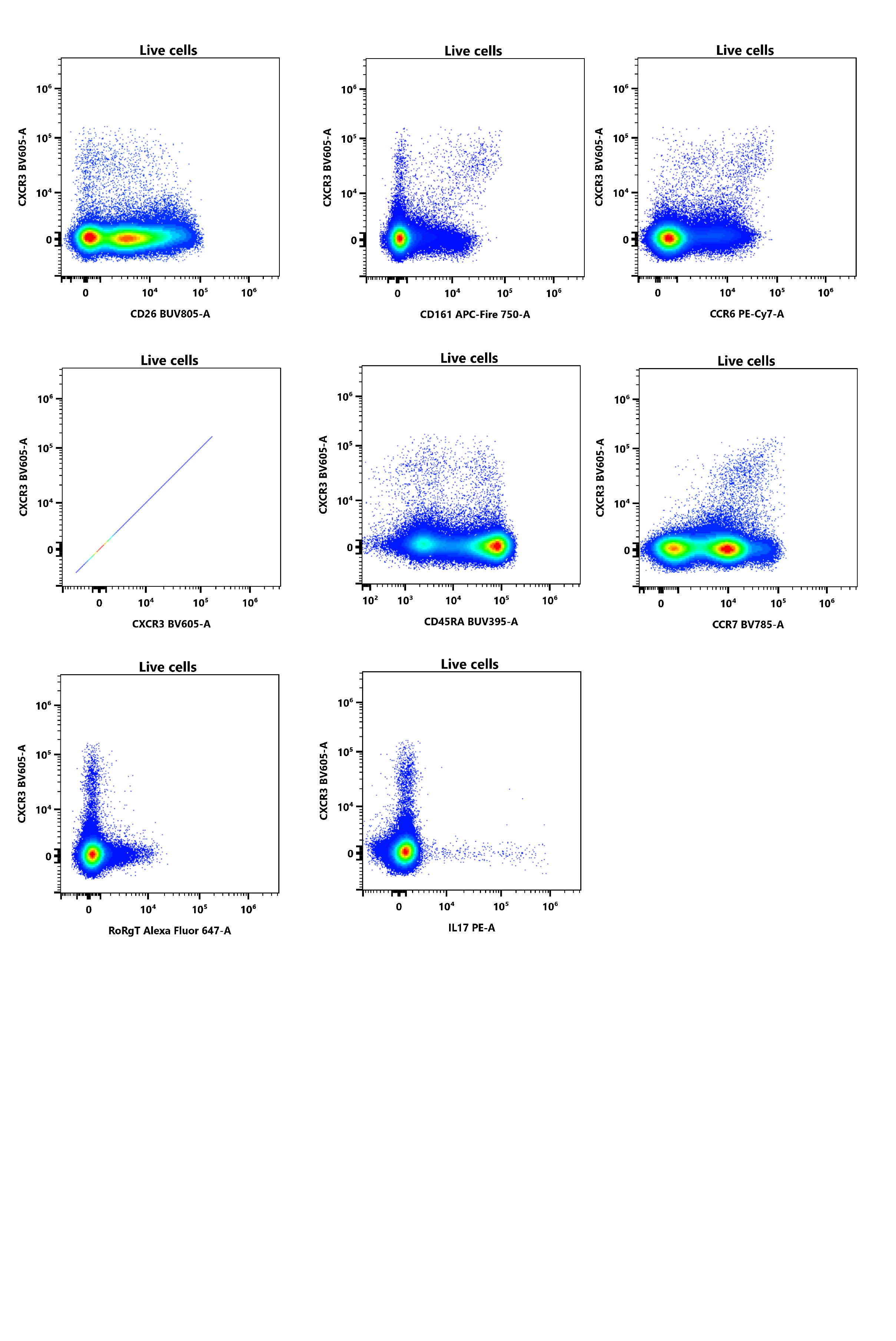
CXCR3

The plots show co-expression of CXCR3 with select T cell markers

#
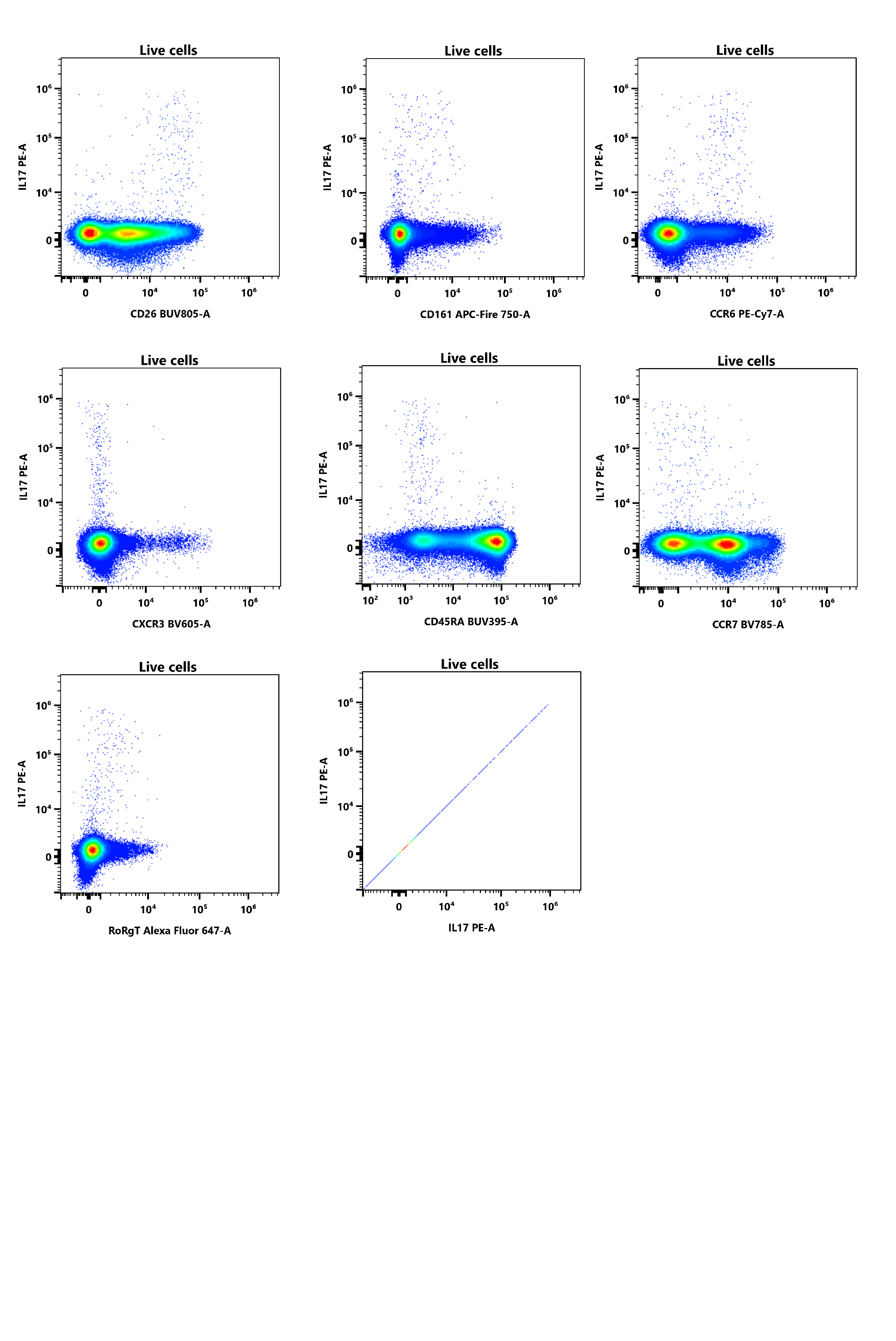
IL17

The plots show co-expression of IL17 with select T cell markers

#
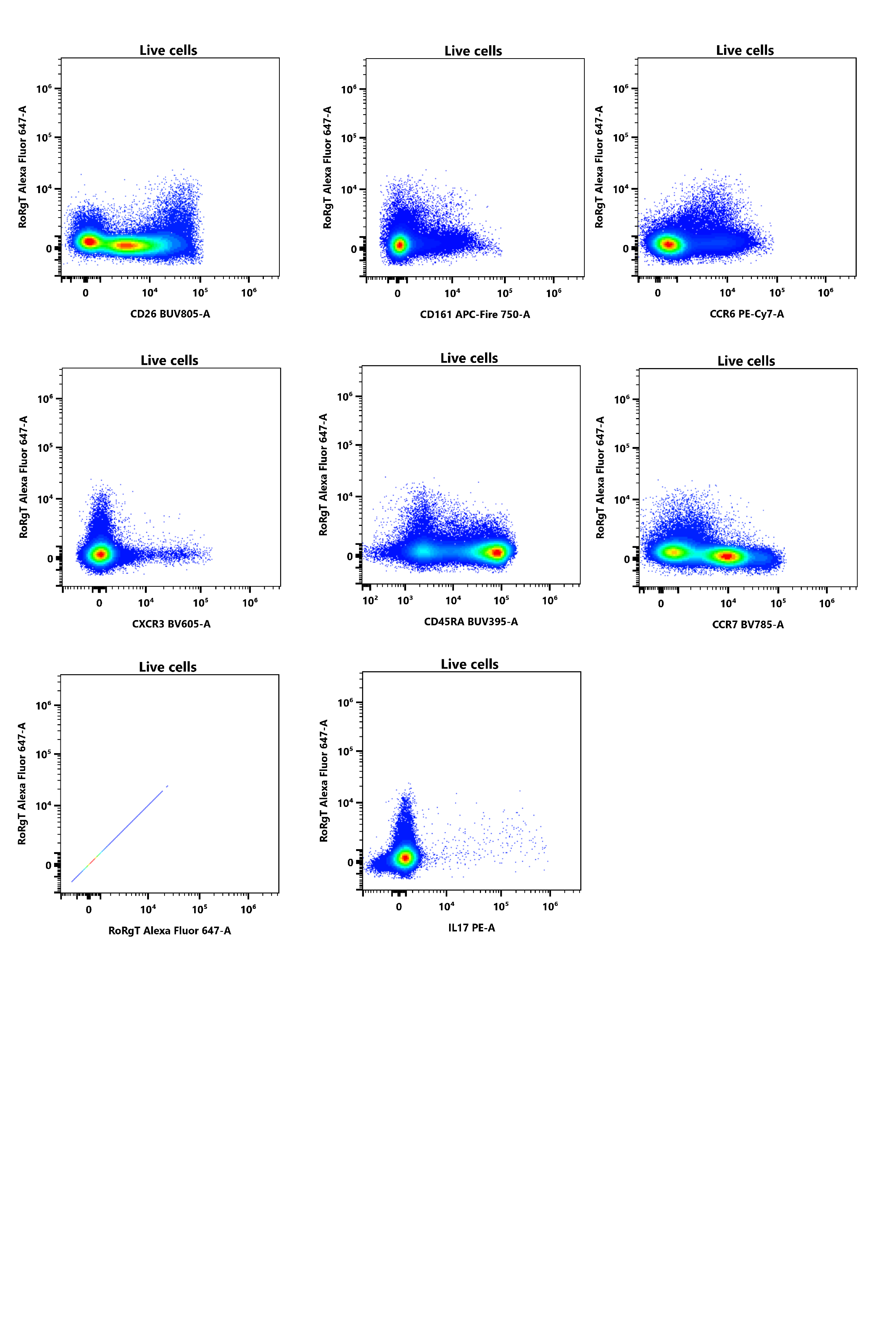
RoRgT

The plots show co-expression of RORγT with select T cell markers

#
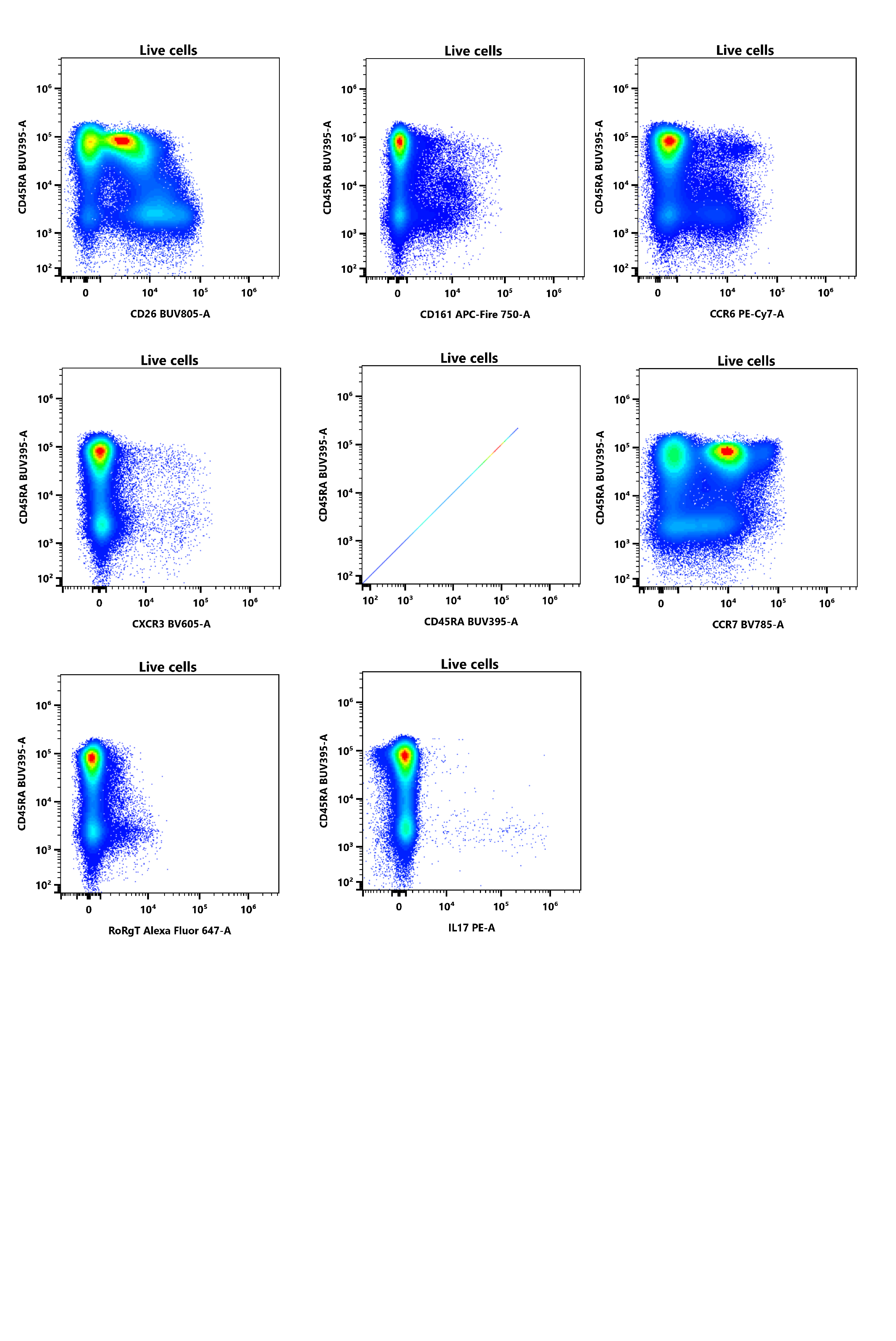
CD45RA

The plots show co-expression of CD45RA with select T cell markers

#
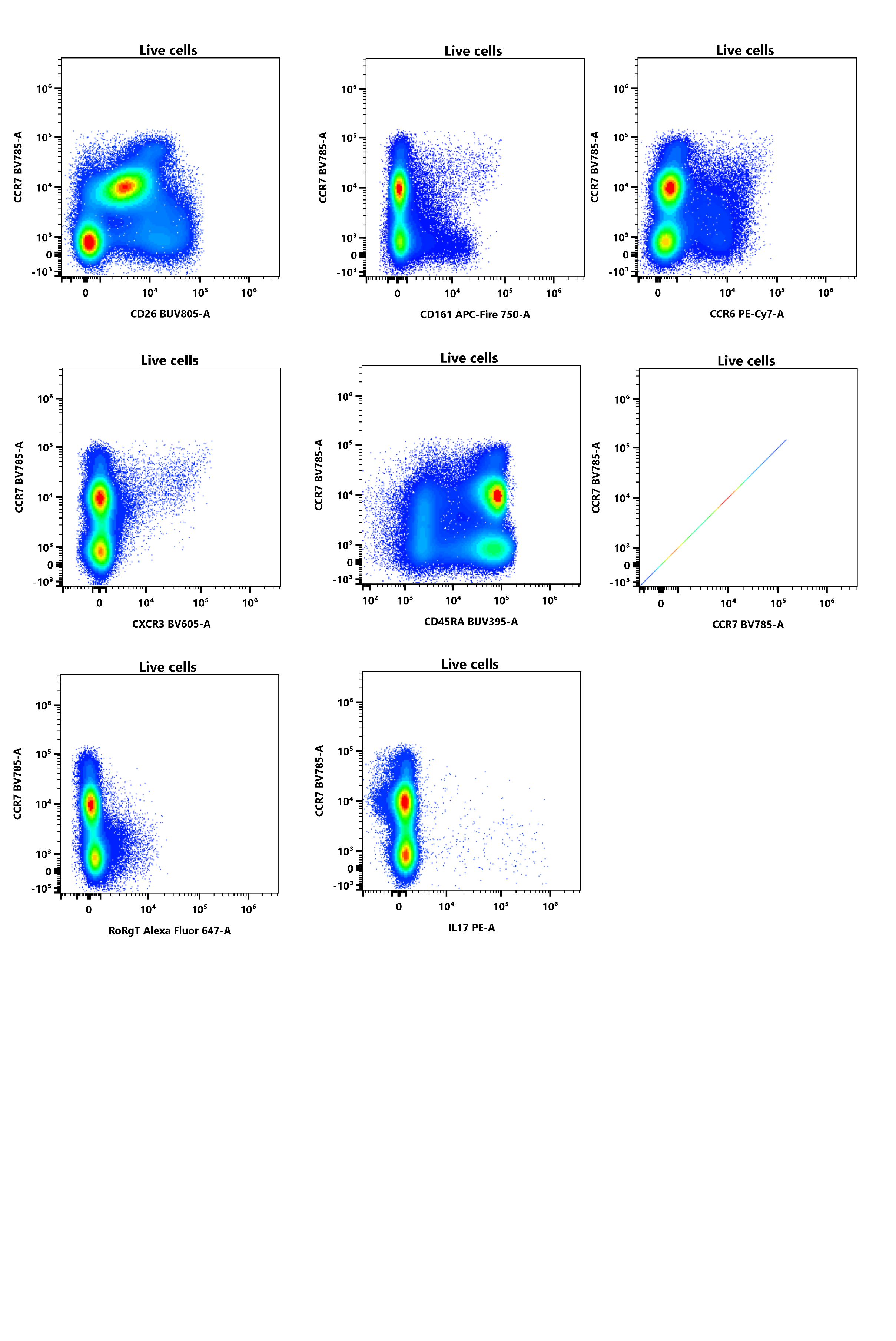
CCR7

The plots show co-expression of CCR7 with select T cell markers
