# Supplementary material for: Increased circulating Th17 cells and altered CD4 T cell maturation and differentiation in active tuberculosis with type 2 diabetes: a pilot study
Source: Front Immunol. 2025 Sep 9;16:1637868. doi: 10.3389/fimmu.2025.1637868 (PMC12454991; doi:10.3389/fimmu.2025.1637868)
Supplement: Supplementary file 2 [file Table1.docx]

**Increased circulating Th17 cells and altered CD4 T cell maturation and differentiation in active tuberculosis with type 2 diabetes: a pilot study**

Paul Ogongo^1*^, Yoscelina E. Martinez-Lopez^2^, Anthony Tran^1^, Cecilia S. Lindestam Arlehamn^3,4^, Alessandro Sette^3,5^, Ilse A. Dominguez-Trejo^2^, Lizette Garza^6^, America M. Cruz-Gonzalez^7^, Raul Loera-Salazar^8^, Javier E. Rodríguez-Herrera^8^, Genesis P. Aguillón-Durán^2,9^, Esperanza M. Garcia-Oropesa^9^, Joel D. Ernst^1^, Blanca I. Restrepo ^2,9,10^


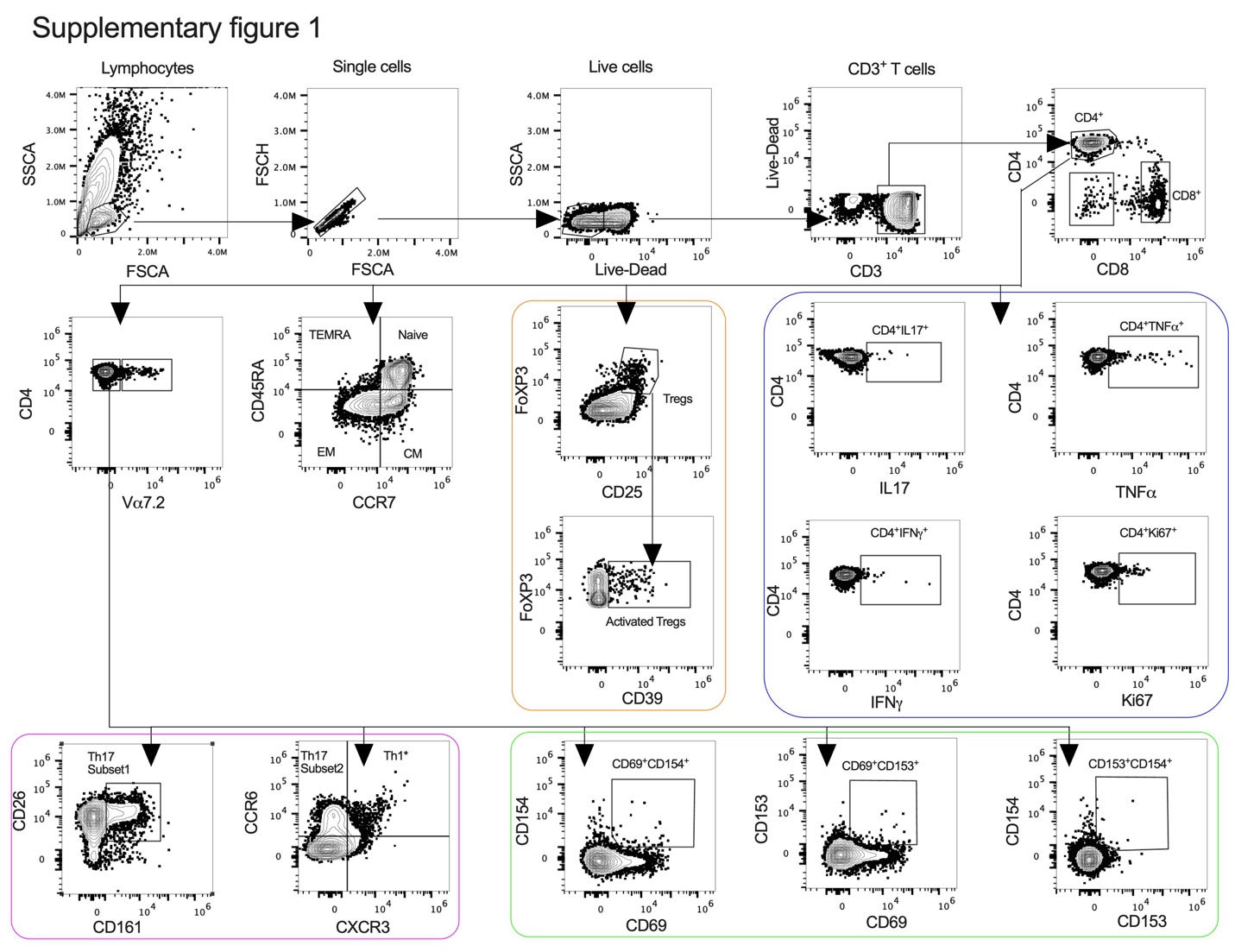


**Supplementary Figure 1: Gating strategy for the characterization of CD4 T cells.** Cells were acquired by spectral flow cytometry followed by unmixing and autofluorescence correction in SpectoFlo v3. T cell subset identification was performed using Flowjo v10. Gating of cells with no antigen stimulation is shown.

**Supplementary Figure 2: T2D does not alter CD8 T cell maturation state but is associated with an increase in circulating Tc17 cell subsets in TB.** CD8 T cells from cryopreserved PBMC without antigen stimulation were analyzed for the expression of CD26, CD161, and CCR6 to identify circulating CD8^+^Va7.2^-^ cytotoxic T cells (Tc17) (**A**). Increased frequency of Tc17 subset 1 (CD8^+^Vα7.2^-^CD26^+^CD161^+^); Tc17 subset 2 (CD8^+^Vα7.2^-^CCR6^+^CXCR3^-^) and Tc17* (Tc1/Tc17: CD8^+^Vα7.2^-^CCR6^+^CXCR3^+^) in TB-T2D compared to TB. In (**B**), maturation state analysis was based on expression of CD45RA and CCR7 as Naive: CD45RA^+^CCR7^+^; Central Memory (CM): CD45RA^-^CCR7^+^; Effector Memory (EM): CD45RA^-^CCR7^-^; Terminally differentiated effector (TEMRA): CD45RA^+^CCR7^-^. Statistics: Wilcoxon rank-sum test.

**Supplementary Figure 3: IL17-producing Mtb-antigen specific cells are enriched in Th17 subset 1 (CD26^+^CD161^+^) than Th17 subset 2 (CCR6^+^CXCR3^-^).** 1x10^6^ live cells were stimulated with Mtb300 megapool antigen (2μg/ml) for a total of 20 hours in the presence of costimulatory antibodies anti-CD28 and anti-CD49d, with Golgi Stop and Golgi Plug added 2 hours after the start of stimulation. Cytokine production was determined by intracellular staining, and cells were analyzed by spectral flow cytometry. Values shown after subtracting the no-antigen stimulation control; a value of 0.001 was assigned for donors in whom there was no response above background. As reported in the main text, there was no difference in the frequency of IL17 between TB-T2D and TB; therefore, the disease groups were combined for this analysis, which is based on Th17 cell subsets, not disease state. Cyan (TB), Brown (TB-T2D) Statistics: Wilcoxon rank-sum test.

**Table S1. Glucose-lowering medications used by Type 2 diabetes in the past month ^1^**

| **Any medication** | 11 (92%) |
| --- | --- |
| **Number of medications** |  |
| No medication | 1 (8%) |
| 1 medication | 4 (33%) |
| 2+ medications | 7 (58%) |
| **Insulin** | 1 (8%) |
| **Metformin** | 10 (83%) |
| **Glybenclamide** | 6 (50%) |
| **Metformin + glybenclamide** | 5 (42%) |

^1^ Data expressed as n (column %) for categorical or median (interquartile range) for continuous variables

**Table S2. Dyslipidemias and complete blood counts with differential by T2D status ^1^**

| **Lipid concentration** | **n** | **TB-No T2D** | **n** | **TB-T2D** | **p value** |
| --- | --- | --- | --- | --- | --- |
| Total cholesterol (mg/dl) | 11 | 125 (42) | 12 | 160.5 (49) | 0.109 |
| HDL cholesterol (mg/dl) | 11 | 40 (12) | 12 | 35.5 (10.5) | 0.758 |
| LDL cholesterol (mg/dl) | 11 | 69.5 (34.7) | 12 | 94 (45.4) | 0.230 |
| Triglycerides (mg/dl) | 11 | 82 (29) | 12 | 119.5 (36) | **0.001** |
| **Complete blood counts and ratios** |  |  |  |  |  |
| Hemoglobin (g/dL) | 9 | 12.5 (1.9) | 12 | 12.1 (3.3) | 0.803 |
| Platelet (10e3/ul) | 9 | 449 (55) | 12 | 488.5 (196) | 0.915 |
| White blood cells (10e3/ul) | 9 | 11.4 (2.9) | 12 | 9.5 (2) | 0.270 |
| Neutrophils (10e3/ul) | 9 | 7.9 (1.4) | 12 | 6.2 (2.4) | 0.145 |
| Lymphocytes (10e3/ul) | 9 | 1.7 (0.2) | 12 | 1.8 (0.3) | 0.337 |
| Monocytes (cells/uL) | 9 | 0.8 (0.4) | 12 | 0.6 (0.2) | 0.189 |
| Eosinophils (10e3/ul) | 9 | 0.2 (0.1) | 12 | 0.1 (0.2) | 0.319 |
| Monocytes:Lymphocyte ratio | 9 | 0.5 (0.3) | 12 | 0.3 (0.1) | 0.166 |
| Neutrophil:Lymphocyte ratio | 9 | **5.1 (1.7)** | 12 | 3.5 (1.6) | **0.051** |

^1^ Data expressed as median (interquartile range) with analysis by Wilcoxon rank sum test.

Abbreviations: T2D=diabetes; HDL-high density lipoprotein; LDL=low density lipoprotein.

**Table S3. TB signs and symptoms and duration, by type 2 diabetes status ^1^**

| **Signs and symptoms and duration** | **n** | **TB-No T2D** | **n** | **TB-T2D** | **p value ^2^** |
| --- | --- | --- | --- | --- | --- |
| Chest pain | 11 | 7 (63.6%) | 12 | 5 (41.7%) | 0.414 |
| Chest pain duration (days) | 10 | 30 (86) | 11 | **0 (20)** | 0.103 |
| Cough | 11 | 11 (100%) | 12 | 12 (100%) | 1.000 |
| Cough duration (days) | 11 | 90 (90) | 12 | **120 (70)** | 0.108 |
| Fever or chills | 11 | 6 (54.5%) | 12 | **10 (83%)** | 0.193 |
| Fever or chills duration (days) | 9 | 7 (18) | 12 | **30 (53)** | 0.134 |
| Hemoptysis | 11 | 4 (36.4%) | 12 | 5 (42%) | 1.000 |
| Hemoptysis duration (days) | 8 | 0 (3) | 10 | 0.5 (4) | 0.849 |
| Dramatic weight loss duration (days) | 9 | 90 (60) | 12 | 60 (61.5) | 0.858 |
| Productive cough duration (days) | 11 | 30 (75) | 12 | **60 (45)** | 0.181 |
| Specify other TB symptom | 11 | 2 (18.2%) | 12 | **6 (50%)** | 0.193 |

^1^ Data expressed as n (column %) for categorical or median (interquartile range) for continuous variables; ^2^ Chi-square for categorical variables with Yates correction and Wilcoxon rank sum test for continuous variables.
